# Diagnostic and therapeutic options in recurrent implantation failure

**DOI:** 10.12688/f1000research.22403.1

**Published:** 2020-03-25

**Authors:** Sarah Moustafa, Steven L. Young

**Affiliations:** 1 Division of Reproductive Endocrinology, Department of Obstetrics and Gynecology, University of North Carolina at Chapel Hill, Chapel Hill, NC, USA

**Keywords:** Recurrent implantation failure, Assisted reproductive technology, in vitro fertilization, Embryo implantation, implantation, pregnancy, endometrium

## Abstract

Recurrent implantation failure (RIF) is an uncommon, imprecisely defined clinical disorder characterized by failure to achieve pregnancy after repeated embryo transfers. The diverse etiologies and incomplete understanding of RIF provide significant diagnostic and therapeutic challenges to patients and providers. Careful clinical evaluation prior to assisted reproduction can uncover many treatable causes, including thyroid dysfunction, submucosal myomas, and tobacco use. The more-subtle causes often require a more-targeted assessment. Undetected, small polyps or small areas of intrauterine synechiae are relatively common and easily treated contributors to RIF. Molecular and cellular abnormalities pose a greater therapeutic challenge. Putative causes of RIF, including progesterone resistance, shifted window of receptivity, decreased integrin expression, and immunologic disturbances, should be considered in the evaluation of a patient with otherwise unexplained RIF. It may also be true that a more complex and standardized definition of RIF would be helpful in these cases. In this paper, we review the diagnostic and therapeutic approaches to RIF, with emphasis on disorders of endometrial receptivity.

## Introduction

Embryo implantation is a delicately coordinated event, relying on multicomponent, bidirectional signaling between the embryo and endometrium ^1, 2^. While embryonic euploidy is one of the greatest determinants of successful conception, human studies suggest that less than 60% euploid embryos result in an ongoing pregnancy ^3^. A number of different lines of evidence demonstrate that normal endometrium is an important part of the implantation process ^4, 5^. This further supports an independent and critical endometrial component for success.

In cases when a good-quality embryo fails to result in pregnancy, an endometrial cause is often suspected but difficult to ascertain in the clinical environment. When good-quality embryos repeatedly fail to implant, a condition known as recurrent implantation failure (RIF), endometrial pathologies are often present. Although there is no universally agreed upon definition, RIF is often defined as the failure to achieve clinical pregnancy after the transfer of four or more good-quality embryos. Many propose alternate cutoffs taking into account maternal age or known euploidy, with consideration for applying the definition to two or more failed embryo transfers in appropriate populations ^6^. A study by Koot *et al*. surveying 118 patients with RIF, defined by failure to conceive after three embryo transfers, found that 49% of respondents ultimately achieved live birth in a 5.5-year follow up period ^7^. While this represents a promising prognosis overall, approximately 50% of RIF patients may ultimately not complete their family goals. A better understanding of the underlying issues is necessary to improve outcomes.

The establishment of endometrial receptivity is primarily coordinated by estrogen and progesterone, by way of direct and indirect transcriptional and translational regulation, leading to changes in function of all endometrial cell types, including glandular and luminal epithelium, stroma, resident immune cells, and endothelium ^8^. Implantation potential is directed by both embryonic factors (euploidy, expression of critical adhesion molecules, trophectoderm differentiation, and adequate invasion) ^9^ and endometrial receptivity.

Estrogen stimulates endometrial proliferation, with resulting thickness directly correlated to success in assisted reproduction ^10^. Estrogen also induces an increase in progesterone receptor expression, enabling the necessary actions of progesterone for the establishment of the “window of receptivity”. These actions include inducing the production of key molecules that promote embryo attachment, such as endometrial integrins ^11, 12^. Disruptions of estrogen and progesterone action have repeatedly demonstrated impacts on pregnancy rates in assisted reproduction. The duration of progesterone exposure is responsible for timing the opening (and closing) of the window of receptivity ^13^. The window of receptivity itself is correlated with a molecular signature and tightly controlled inflammatory response, responsible for coordinating the attachment of an embryo, a permissive state for invasion of this semi-foreign entity, and establishment of adequate vascular supply necessary to nurture a healthy pregnancy ^14^.

Structural components such as polyps, submucosal myomas, intrauterine synechiae, and uterine septums have demonstrated detrimental effects on successful implantation by poorly defined mechanisms, very likely involving these processes ^15^. Generalized conditions, such as endometriosis, have demonstrated effects on various individual components of implantation. Key studies ^16– 18^ demonstrated endometriosis-driven progesterone resistance, disrupting events downstream of progesterone signaling. Meanwhile, other studies have highlighted the impacts of endometriosis-driven inflammatory markers disrupting critical pathways ^19^ for decidualization ^20^, tolerance ^21^, and successful implantation ^22^.

Other systemic disruptions may have a more occult impact on the endometrium. Obesity, for instance, has been associated with altered endometrial gene expression and reduced pregnancy rates in a dose-dependent fashion ^23, 24^. While some of these pathologies may be obvious in a patient with RIF, many patients do not demonstrate clear etiology on routine testing. We intend to review additional evaluations that may be indicated, specifically with regard to the endometrium, and proposed therapies in the literature.

## Evaluations to consider in a patient with recurrent implantation failure

Patients undergoing *in vitro* fertilization (IVF) have ongoing ultrasound evaluation of the endometrium. As noted above, an endometrium that does not sufficiently thicken predicts a lower chance of embryo implantation. It may also suggest the possibility of additional pathology, such as intrauterine synechiae. However, many patients with RIF exhibit normal endometrial thickness and may have other occult processes implicated. Complete evaluation would require both hysteroscopy and endometrial sampling, as discussed below.

### Diagnostic hysteroscopy

Most, if not all, centers utilize at least one form of uterine cavity evaluation prior to embryo transfer. Hysterosalpingogram (HSG), saline-infusion sonography (SIS), and hysteroscopy are all accepted tools for cavity assessment. While hysteroscopy is widely regarded as the gold standard, it is not often deployed as a first-line method because of higher costs and equipment needs, even if performed in an ambulatory setting. However, the incidence of uterine pathology in women undergoing IVF has been reported to be as high as 40% ^25^. Additionally, the use of hysteroscopy in patients with prior failed transfers was associated with an increase in clinical pregnancy, whether or not pathology was detected, perhaps owing to a benefit of endometrial injury and repair ^26^. One small study showed that approximately 43% of patients with a previously normal uterine cavity evaluation were found to have abnormality on hysteroscopy ^27^. While previously missed pathology may be minor in size, studies have suggested significant fecundability improvements with removal of polyps or intrauterine adhesive disease ^15^ and that molecular changes may impair implantation, even if size or location does not ^28^. Furthermore, hysteroscopy may raise suspicion of one culprit of RIF: chronic endometritis (CE, see below). Features such as micropolyps and hyperemic or edematous endometrium may be identified on hysteroscopy, and these may raise suspicion for otherwise subclinical CE. Targeted biopsy allows confident CE diagnosis (with caveats), and studies suggest successful identification and treatment may improve pregnancy rates ^29^.

### Endometrial sampling

Targeted endometrial sampling at the time of hysteroscopy or more general sampling in the office allows the identification of subclinical or clinically suspected CE. While some studies have shown the incidence of CE is low in a general infertility population ^30^, multiple studies have demonstrated a high incidence of CE (30%) patients with RIF ^29, 31^, and it must be considered in the evaluation of unexplained RIF. Cicinelli *et al*. demonstrated that adequate treatment of CE in this population resulted in significantly higher pregnancy rates than in patients who had persistent CE after therapy. Additionally, endometrial biopsy may provide value in the diagnosis of other endometrial pathology such as polyps (whether or not this is performed with hysteroscopy). Unfortunately, diagnostic criteria for endometritis have remained a subject of debate for decades, limiting the precision of this diagnosis ^32, 33^. Despite the uncertainty, CE is correlated with reproductive failure, and the relatively benign treatment with antibiotics is likely beneficial to identified cases ^29^. Polyp identification on an undirected biopsy can lead to hysteroscopic removal, and a portion of the same endometrial sample can be used for specialized endometrial testing (see below). For these reasons, we strongly support the use of endometrial sampling as part of the evaluation of all RIF patients.

### BCL6 testing

Increased BCL6 expression in the endometrium has been implicated in patients with unexplained or endometriosis-associated infertility ^34, 35^. While the vast majority of patients with endometriosis exhibited aberrant BCL6 expression, it cannot be ruled out that abnormal expression may occur in the absence of evident endometriosis. Regardless of fertility diagnosis, over-expression of this transcriptional gene repressor has been linked to progesterone resistance by disrupting early and critical P4 signaling ^16^. A prospective study in patients with unexplained infertility by Almquist *et al*. showed a live birth rate following IVF of 11.5% versus 58% in patients with and without elevated BCL6, respectively ^35^. Furthermore, Likes *et al*. found that treating these patients with GnRH agonist or surgical management of endometriosis lesions significantly improved pregnancy rates ^36^. As such, it is reasonable to consider BCL6 testing in patients with otherwise unexplained RIF.

### Endometrial receptivity analysis and related assays

Many studies have demonstrated different genomic signatures between pre-receptive and receptive endometrium ^37, 38^. Similarly, an altered endometrial transcriptome has been implicated in patients with RIF, suggesting that these patients may fail to achieve the necessary molecular signature for receptive endometrium ^39^. These approaches are based on the premise that the window of receptivity is present for a highly specific period of time defined by hormone exposures. In 2011, Diaz-Gimeno *et al*. published results of the Endometrial Receptivity Analysis (ERA), including 238 genes, which could accurately determine endometrial dating ^40^. Alonso *et al*. ^41^ utilized this analysis to show that approximately 25% of patients with RIF had an altered window of implantation (WOI), with the correct gene signature occurring before or after the expected time of 5.5 days of progesterone exposure. These results argued for personalized embryo transfer (pET) timing. While utility has not yet been fully demonstrated in a generalized population, the altered genomic signature in patients with RIF ^42, 43^ may be a therapeutic opportunity. The earlier works of Alonso *et al*. have been validated in a recent study by Tan *et al*., supporting a higher incidence of an altered WOI in RIF ^44^. However, owing to limited size and follow up, both studies were unable to conclude clear and significant benefit from ERA and pET in RIF. Observational studies suggest a prevalence of abnormal ERA testing in about 25% of patients with RIF (as noted above) but also in about 12–15% of control groups ^41, 45^. Relative abnormal test rate in the control group suggests the existence of false positive ERA testing. In the largest study examining the utility of ERA, performed in patients undergoing their first IVF cycle, the authors showed preliminary results with pregnancy rate improving in pET, but not live birth rates ^46^. While the premise and data derived from ERA testing are compelling, further studies are needed to understand if there is a refined clinical application that would result in consistent outcome improvements.

Currently, the ERA test is available from a single diagnostic company, but competitors are beginning to emerge and will need to be validated and compared to existing technology ^47^. An earlier alternative to the ERA which may be considered is immunohistochemistry evaluation of cyclin E and p27, which exhibit progesterone exposure-dependent alterations in localization ^48^. Head-to-head comparisons of commercially available methods have not been performed.

## Management options for unexplained recurrent implantation failure

So far, we have reviewed testing for additional pathologies possibly implicated in RIF, with known management options. However, a significant portion of RIF patients will not yield definitive etiology. Many studies have examined experimental therapies in these “unexplained” RIF patients.

### Diagnostic and therapeutic laparoscopy

Once a standard component of an infertility evaluation, diagnostic laparoscopy is now seldom performed for the sole purpose of diagnosing causes of infertility. Given the increased success of IVF after prolonged pituitary downregulation in women with endometriosis ^49^ or surgery ^50^, with a likely benefit to embryo implantation, the use of laparoscopy might be considered to identify and treat otherwise unrecognized endometriosis. However, many have argued that the number needed to treat is prohibitive to routine laparoscopy, with the American Society for Reproductive Medicine (ASRM) citing, “the number of laparoscopies that need to be performed to gain one additional pregnancy is actually 40” ^51^. However, a valid argument can be made that women with significant dysmenorrhea or with abnormal BCL6 ^36^ or miRNA ^52^ testing have a dramatically increased chance of endometriosis, leading to a much more favorable number needed to treat for surgical management and/or prolonged pituitary downregulation.

### Intrauterine human chorionic gonadotropin

Human chorionic gonadotropin (hCG) is produced by the trophectoderm and aids in embryonic invasion ^53^. The hCG receptor is highly expressed in the endometrium and has been found to coordinate cytokine secretion during the receptive period ^54^. As a result, instillation of intracavitary hCG immediately preceding embryo transfer has been proposed and attempted in order to improve pregnancy rates in assisted reproduction. Strug *et al*. found increased expression of estrogen and progesterone receptors in the endometrium following hCG instillation, administered after stimulation in oocyte donors, as well as increase in other targets with roles in implantation, such as C3 and NOTCH1 ^55^. Notably, a favorable shift in ERA testing was not appreciated.

A 2018 Cochrane review of 17 randomized controlled trials (RCTs) concluded that while exogenous hCG may provide value in the transfer of cleavage-stage embryos, no benefit was seen at the blastocyst stage ^56^. However, the preponderance of RCTs included in the Cochrane review were studies of exogenous hCG during exclusively fresh or fresh and frozen transfer cycles. This population would have received systemic hCG 7 days prior and is not applicable to a frozen transfer cycle, wherein exposure to hCG would be limited to the study intervention and the local production of hCG by the implanting embryo. Furthermore, it is worth noting that the majority of studies did not limit the study population to patients with prior implantation failure. Interestingly, several studies that specifically examined the role of this intervention in RIF patients found significant improvement in pregnancy rates ^57, 58^. A recent systematic review concluded that hCG improved clinical pregnancy rates and live birth rates while reducing miscarriage ^59^. These studies encompass a small number of subjects, but existing data suggest that hCG use merits further investigation in RIF patients.

### Endometrial injury or “scratch”

The idea of endometrial injury to improve implantation has been used as early as 2003, with Barash *et al*. showing benefit in patients with prior failed transfers ^60^. The concept is based on inciting an acute inflammatory reaction, followed by repair, resulting in the release of cytokines and growth factors known to promote implantation ^61^. Optimal timing for positive effects of the procedure was the cycle preceding transfer, with same-cycle scratch possibly detrimental to pregnancy rate ^62^. However, contrary to prior studies investigating impact on day of retrieval, a more recent study did find improved pregnancy rates with endometrial injury performed during menstruation of the same cycle (64% versus 48%, *P* = 0.023) ^63^. Also, in contrast to previous studies, Tang *et al*. examined a RIF population rather than scratch effect on primary cycles. It is unclear if the difference in outcome is driven by the population or timing of endometrial scratch. The quality of overall evidence and global applicability of this method have been questioned, particularly because of the heterogeneity of RCTs ^64, 65^. Potdar et al. ^66^ and Vitagliano *et al*. ^61^ published comprehensive reviews and meta-analyses concluding that this intervention does impart significant benefit for women with prior implantation failure. However, a large RCT of 1,364 women published in 2019 showed no advantage to endometrial injury in the generalized population or in a subgroup of patients who had two or more failed transfers ^67^. Discussion of this paper has yielded a variety of polarized conclusions ^68^. At this time, the low-risk procedure of endometrial injury via endometrial biopsy and/or hysteroscopy is useful for diagnostic purposes, while a clearer understanding of the pathophysiology of RIF and effects of endometrial injury will be necessary to better understand a possible therapeutic role and possibly refined patient selection in RIF.

### Granulocyte colony-stimulating factor

Granulocyte colony-stimulating factor (GCSF) has been proposed to improve endometrial thickness as well as treat RIF. The data on the use of GCSF to improve endometrial thickness have been conflicting. While several groups have produced evidence supporting improved endometrial thickness and pregnancy rates ^69, 70^, these findings are not uniform ^71^ and the hypothesis that GCSF promotes endometrial thickness and pregnancy rates in women with thin endometrium remains to be evaluated in a blinded, randomized fashion. Without further data, we interpret the evidence as not supporting the use of GCSF to improve endometrial thickness.

The use of GCSF for RIF in patients, independent of endometrial thickness, has the support of a single unblinded RCT that demonstrates significantly improved implantation and live birth rates ^72^. In this study, 112 patients were randomized to receive subcutaneous GCSF 1 hour prior to day 3 embryo transfer or no intervention, resulting in an adjusted odds ratio of 2.63 (1.09–6.96) for implantation rate. The findings are consistent with at least one other recent retrospective cohort study in an RIF population ^73^. While repeated demonstration in subsequent RCTs would support more widespread use of this intervention, existing evidence suggests minimal harm and potential benefit to this therapy in indicated patients with unexplained RIF.

### Platelet-rich plasma

Platelet-rich plasma (PRP) is an experimental intervention, initially geared at addressing poor proliferation and suboptimal endometrial thickness by promoting tissue regeneration. Several small, observational studies have demonstrated a positive impact of intrauterine infusion of PRP on endometrial thickness and pregnancy rates ^74, 75^. The largest available RCT was performed by Nazari *et al*. in 138 RIF patients and supported a possible benefit ^76^. Another study investigated the use of PRP in patients with normal or optimal endometrial thickness and did not note a statistically significant improvement in pregnancy rates ^77^. Most existing studies are limited by small numbers, lack of randomization, and suboptimal control groups. Further research is warranted, but we interpret the evidence in support of the use of PRP for RIF as too limited for generalized application.

### Letrozole

As mentioned in previous sections, an important etiology to consider for RIF is altered hormone exposure or receptor expression in the endometrium, with consequently altered expression of key molecules for receptivity, such as ανβ3 integrin and leukemia inhibitory factor (LIF). Occult endometriosis is considered a possible etiology for RIF and is associated with decreased integrin expression and increased aromatase expression ^78, 79^. One proposed solution to this is the use of an aromatase inhibitor such as letrozole to restore integrin expression. A retrospective study demonstrated an increase in integrin expression as well as ongoing pregnancy rate in patients who were initially integrin negative and treated with letrozole compared to those who were integrin negative and did not receive the intervention ^80^. These results were validated in a more recent retrospective cohort study utilizing both a GnRH agonist and letrozole, with improved live birth rates in RIF patients receiving letrozole (superior to GnRH alone) ^81^. While RCTs are needed to further support the use of this intervention, existing basic and clinical evidence combined with low cost and no evidence of harm support a consideration of letrozole in RIF patients.

### Growth hormone

Growth hormone (GH) expression has been demonstrated in secretory-phase endometrium ^82^ and shown to promote the expression of critical factors for receptivity such as VEGF, LIF, and β3 integrin subunit ^83, 84^. In one RCT, Altmae *et al*. showed an improvement in endometrial thickness, pregnancy, and live birth rates in oocyte donor recipients with RIF treated with GH compared to those who were not ^85^. The data are encouraging, and GH is low risk (other than expense), and thus the data may prompt use in some cases of RIF. Additional studies would be helpful in better understanding the role of this intervention.

### Glucocorticoids

As previously mentioned, cytokines and uterine natural killer cells have important roles in successful implantation ^86^, but excessive and altered inflammatory signaling has long been suspected in implantation failure and recurrent pregnancy loss. This conceptual paradigm led to widespread use of glucocorticoids based on the biologic plausibility of restoring a normal immunologic response in the endometrium to promote healthy embryo implantation. Despite this general rationale, many small RCTs in general IVF populations have shown no clinical improvements with glucocorticoid treatment. Larger reviews and meta-analyses have similarly failed to show a benefit. As a result, ASRM guidelines ^87^ currently recommend against the routine use of glucocorticoids to improve implantation rates. It is important to note, however, that data specific to RIF are lacking. Some data suggest that immune-modulation in patients without auto-antibodies or exaggerated cytotoxic activity may provide benefit ^88, 89^. Any benefit of glucocorticoids remains unclear, and there are rare, but clinically significant, side effects, relegating their use to research or, possibly, as a last resort in select cases with clinical and histological evidence of excessive inflammation without chronic infection. Similarly, other immune-modulators, such as IVIG and intralipids, may also be considered as a last resort in patients with punitive immunologic disorders ^90, 91^. However, these data are significantly limited, and treatments are expensive.

## Summary

RIF is a clinical problem, often with poorly defined criteria and underlying etiology. Despite the lack of full consensus, studies strongly implicate problems affecting the endometrial cavity, many of which are amenable to treatment.
